# Generation of a Mouse Model to Study the Noonan Syndrome Gene *Lztr1* in the Telencephalon

**DOI:** 10.3389/fcell.2021.673995

**Published:** 2021-06-16

**Authors:** Mary Jo Talley, Diana Nardini, Nisha Shabbir, Lisa A. Ehrman, Carlos E. Prada, Ronald R. Waclaw

**Affiliations:** ^1^Graduate Program in Molecular and Developmental Biology, Cincinnati Children’s Hospital Research Foundation, University of Cincinnati College of Medicine, Cincinnati, OH, United States; ^2^Division of Experimental Hematology and Cancer Biology, Cincinnati Children’s Hospital Medical Center, Cincinnati, OH, United States; ^3^Division of Human Genetics, Cincinnati Children’s Hospital Medical Center, Cincinnati, OH, United States; ^4^Department of Pediatrics, University of Cincinnati College of Medicine, Cincinnati, OH, United States; ^5^Division of Developmental Biology, Cincinnati Children’s Hospital Medical Center, Cincinnati, OH, United States

**Keywords:** *LZTR1*, Noonan syndrome, MAPK, oligodendrocyte, astrocyte

## Abstract

The leucine zipper-like transcriptional regulator 1 (*Lztr1*) is a BTB-Kelch domain protein involved in RAS/MAPK pathway regulation. Mutations in *LZTR1* are associated with cancers and Noonan syndrome, the most common RASopathy. The expression and function of *Lztr1* in the developing brain remains poorly understood. Here we show that *Lztr1* is expressed in distinct regions of the telencephalon, the most anterior region of the forebrain. *Lztr1* expression was robust in the cortex, amygdala, hippocampus, and oligodendrocytes in the white matter. To gain insight into the impact of *Lztr1* deficiency, we generated a conditional knockout (cKO) restricted to the telencephalon using *Foxg1^*IREScre/*+^. Lztr1* cKOs are viable to postnatal stages and show reduced *Lztr1* expression in the telencephalon. Interestingly, *Lztr1* cKOs exhibit an increase in MAPK pathway activation in white matter regions and subsequently show an altered expression of stage-specific markers in the oligodendrocyte lineage with increased oligodendrocyte progenitor cells (OPCs) and decreased markers of oligodendrocyte differentiation. Moreover, *Lztr1* cKOs also exhibit an increased expression of the astrocyte marker GFAP. These results highlight the generation of a new mouse model to study *Lztr1* deficiency in the brain and reveal a novel role for *Lztr1* in normal oligodendrocyte and astrocyte development in the telencephalon.

## Introduction

*LZTR1* was originally identified as one of the candidate genes deleted in some 22q11.2 deletion syndrome patients (Kurahashi et al., 1995). It is a unique member of the BTB-Kelch superfamily due to its localized expression to the golgi network *via* the C-terminal BTB/POZ domain (Nacak et al., 2006). *LZTR1* is also thought to be a tumor suppressor as loss-of-function mutations have been associated with schwannomatosis and glioma (Frattini et al., 2013; Piotrowski et al., 2014). The function of *Lztr1* has been elusive, but the protein contains six N-terminal Kelch motifs (Nacak et al., 2006) which are proposed to mediate protein–protein interactions and are associated with Cullin3 ubiquitination complexes (Shi et al., 2019). *LZTR1* mutations have also been associated with Noonan syndrome (NS), a type of RASopathy affecting multiple organ systems, including growth, craniofacial, cardiovascular, and neurocognitive abnormalities (Yamamoto et al., 2015). Interestingly, mutations in *LZTR1* are associated with both autosomal dominant and biallelic recessive forms of NS. Missense mutations in the Kelch domain that do not affect protein stability are associated with dominant NS, while loss-of-function or inactivating mutations are associated with biallelic recessive NS (Yamamoto et al., 2015; Johnston et al., 2018; Motta et al., 2019). *LZTR1* is the first NS gene described to be inherited in this unique manner. While the NS clinical phenotype can be variable, Johnston et al. (2018) showed examples of early lethality and also cancers associated with the biallelic recessive mutations, which suggests that the *LZTR1* loss-of-function or inactivating mutations might align with a more severe type of NS.

RASopathies, including NF1, NS, Costello syndrome, and cardiofaciocutaneous syndrome, are a group of distinct diseases that are caused by mutations that upregulate the RAS/MAPK pathway (reviewed in Tartaglia and Gelb, 2010). The identification of *LZTR1* mutations with NS implicates *LZTR1* as a regulator of the RAS/MAPK pathway. However, the function of *LZTR1* in the RAS/MAPK pathway is still an active area of research. Recent studies have suggested that *LZTR1* plays a key role in the ubiquitination of RAS proteins (Bigenzahn et al., 2018; Steklov et al., 2018). Indeed *LZTR1* has been referred to as a “RAS killer protein” through the polyubiquitination and degradation of RAS-GTPases, thereby controlling the appropriate activation levels of the RAS/MAPK pathway (Abe et al., 2020). In addition to the regulation of RAS ubiquitination, *LZTR1* has also been suggested to regulate other levels of the RAS/MAPK pathway through phosphorylation of RAF1 or stabilization of RIT1 to impact the activity of the MAPK pathway (Castel et al., 2019; Umeki et al., 2019). Given its association with human disease and the multiple lines of evidence suggesting that *LZTR1* function is key to controlling MAPK pathway activation, the development of new approaches and models to modulate gene function is essential to understand the pathophysiology of *LZTR1*-associated diseases.

Neurocognitive abnormalities are observed in NS patients (Pierpont et al., 2009; Alfieri et al., 2011), making it necessary to understand how gene mutations associated with NS impact the developing brain. While progress continues to be made in determining the molecular role of *LZTR1* in the RAS/MAPK pathway, the impact of *LZTR1* loss, specifically in the brain, has not yet been examined. Heterozygous loss of *Lztr1* in mice (*Lztr1*^+/–^) reproduces some of the craniofacial, cardiovascular, and growth-associated NS phenotypes in the heterozygous state (Steklov et al., 2018). Lethality in *Lztr1^–/–^* mouse embryos (Steklov et al., 2018) prevents the analysis of the biallelic loss-of-function mutations on cellular populations in the postnatal brain. Our study will be useful for understanding *Lztr1* biology by developing a cell type-specific genetic mouse model to determine the impact of *Lztr1* loss of function in the developing brain.

*LZTR1* mutations are associated with NS and 22q11.2 deletion syndrome, both of which show a variety of phenotypes, including neurological dysfunction (Pierpont et al., 2009; Fiksinski et al., 2021). In this study, we examine a role for *Lztr1* in the anterior forebrain, a region associated with higher neural functions, like cognition and emotion (Rubenstein, 2011). Our studies reveal that *Lztr1* is highly expressed in the cortex, amygdala, hippocampus, and white matter regions. Utilizing a conditional knockout strategy restricted to the telencephalon that results in postnatal viability, we have identified a novel role for *Lztr1* in the normal generation of oligodendrocyte progenitor cells (OPCs) and the balanced establishment of oligodendrocyte and astrocyte differentiation markers. Our results suggest that *Lztr1* gene function is crucial for the normal generation of glial cells in the telencephalon.

## Methods

### Animals

The animal protocols for experiments using mice were approved by the Cincinnati Children’s Hospital Medical Center Institutional Animal Care and Use Committee and carried out in accordance with the National Institutes of Health guidelines. *Lztr1^*tm*1a/+^* knock-out mice were first purchased from EMMA/EUCOMM (EM #06794), and the mouse generation strategy was as described in Skarnes et al. (2011). These mice were converted to a lacZ reporter line (*Lztr1^*tm*1b/+^*, also called *Lztr1^*lacZ/*+^*) using EIIA germline cre transgenic mice (JAX#003724). The *Lztr1^*tm*1a/+^* mice were also converted to the conditional allele (*Lztr1^*tm*1c/+^*, also called *Lztr1^*fx*/+^*) using germline Flpase transgenic mice (JAX#005703). *Foxg1^*IREScre/*+^* (JAX #029690) mice were used to delete *Lztr1* in the telencephalon. Note that this cre is inserted downstream of the *FoxG1* coding region in the UTR, preserving *FoxG1* expression (Kawaguchi et al., 2016). The mice obtained from Jax were genotyped from published protocols on the Jax website. The *Lztr1^*fx/*+^* mice have loxP sites flanking exon7 of *Lztr1* variant 1 and were genotyped with the following primers: lztr1-loxP5′: TCTGAGCTCCTGTGTTGCAG and lztr1-loxP3′: GGGGTAATGAGTCGCCTGAG. The WT allele results in a 503-bp product, and the Lztr1-loxP allele results in a 583-bp product. *Lztr1^*lacZ/*+^* was genotyped with the following β-gal-specific primers, β-gal-5′: AACTTAATCGCCTTGCAGCA and β-gal-3′: GTAACCGTGCATCTGCCAGT, that produced a 218-bp product. Embryonic and postnatal brains were collected, fixed, and processed for histology as previously described (Waclaw et al., 2006, 2010; Ehrman et al., 2014). For all the stages analyzed, at least three embryos or postnatal brains were included for each genotype. *Lztr1* conditional knockouts (*Lztr1^*fx/lacZ*^; Foxg1^*IREScre/*+^*) were compared to controls (*Lztr1^+/+^* or *Lztr1^*fx/*+^*).

### Immunohistochemistry and Fluorescence

Primary antibodies were used at the following concentrations: Gt-β-gal (1:1,000, from Biogenesis, #2282), Rbt-GFAP (1:5,000, from DAKO, #Z0334), Rbt-Olig2 (1:1,000, from Millipore-Sigma, #AB9610), Rbt-Nkx2.2 (1:1,000, from Abcam, ab191077), Rbt-Tcf7l2 (1:500, from Cell Signaling, #2569), Gt-Pdgfra (1:500 from R&D Systems, #AF1062), and Rbt-p-ERK1/2 (1:500 from Cell Signaling, #9101 and #4370). *In situ* hybridization was completed as described (Kohli et al., 2018). The plasmids to generate *Plp1* anti-sense probe were provided by Dr. Q. Richard Lu (CCHMC). The Lztr1 anti-sense probe was generated with the following primers that amplify a product 3′ of the loxP deletion: Lztr1-5′: GCTGTGTGCCGGGATAAGA and Lztr1-3′ with T3: ATTAACCCTCACTAAAGG GCTCAGCGCCAACTTATACAC (966-bp probe). The LacZ anti-sense probe was generated with the following primers, LacZ-5′: AACTCGGCTCACAGT ACGC and LacZ-3′ with T3: ATTAACCCTCACTAAAGGAG TAAGGCGGTCGGGATAGT (656-bp probe). The Enpp6 anti-sense probe was generated from the following primers: Enpp6-5′: TAGCATCCTTGCCTGGCTTC and Enpp6-3′ with T3: ATTAA CCCTCACTAAAGG CCGCTCGGCCACTCTTTAAT (417-bp probe).

Brightfield pictures were captured on a Leica DM2500 microscope with either a Leica DFC500 or DMC6200 camera using Leica Acquisition Software (LAS-X). Fluorescent images were captured on a Nikon C2 confocal microscope using Nikon Elements software. Raw images were processed using CellProfiler for quantification, and numbers are presented as cells/mm^2^ or arbitrary intensity units, as indicated by graphs. The labeled cells in P21 or P30 mice were counted in two serial sections in both hemispheres at the indicated levels and magnification for a total of four images per brain. For all quantifications, at least three different controls or experimental samples (cKO) were used. Unless otherwise stated, statistics were performed between control and *Lztr1* cKO using Student’s unpaired *t*-test. Graphs were generated using GraphPad Prism. Images were brightened in Adobe Photoshop equally between control and *Lztr1* cKOs.

## Results

### Expression of *Lztr1* in the Telencephalon

**FIGURE 1:**
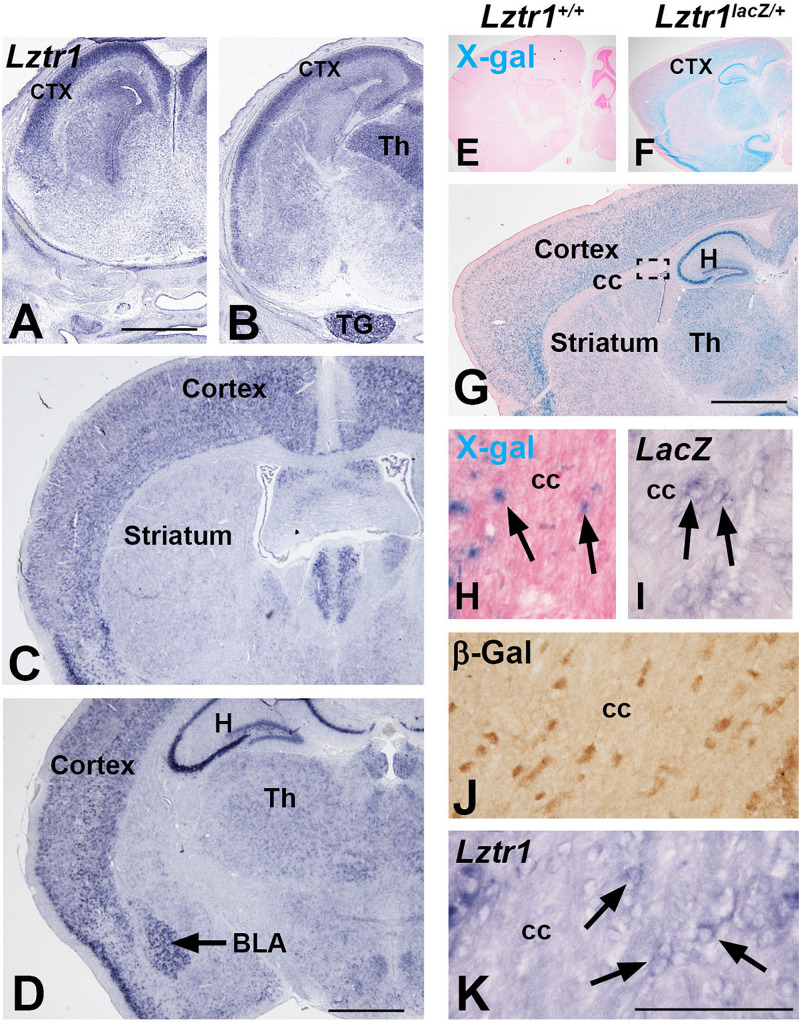
*Lztr1*expression in the developing telencephalon. *Lztr1* gene expression in the cortex and thalamus at both E18.5 **(A,B)** and postnatal (1 month old) stages **(C–K)**. *Lztr1* is also observed in the trigeminal nerve at E18.5 **(B)** and the hippocampus and basolateral amygdala in the postnatal brain (black arrow, **D**). X-gal reaction in postnatal brains shows lacZ reporter activity from the *Lztr1* locus (compare **E** to **F**). Sagittal view of the postnatal forebrain shows that lacZ activity is similar to *Lztr1* gene expression (compare **G** to **C,D**). High magnification of the corpus callosum reveal LacZ-positive cells **(H)**, *lacZ* gene expression **(I)**, and β-gal immunohistochemistry **(J)**. This correlates to the *Lztr1* expression observed in the corpus callosum **(K)**. Scale bars = 1 mm in for **(A–D,G)**. Scale bar is 100 μm for **(H–K)**. ctx, cortex; Th, thalamus; TG, trigeminal nerve; H, hippocampus; BLA, basolateral amygdala; CC, corpus callosum.

*LZTR1* is a human disease gene associated with NS and also deleted in some cases of 22q11.2 deletion syndrome (Kurahashi et al., 1995; Yamamoto et al., 2015; Johnston et al., 2018). Patients of these syndromes show a variety of phenotypes, including developmental delay and neurocognitive dysfunction (Pierpont et al., 2009; Fiksinski et al., 2021). However, little is known about the expression or role of *Lztr1* in the developing brain. Early reports suggest a wide expression of *Lztr1* in a variety of organ systems (Kurahashi et al., 1995; Nacak et al., 2006). We describe here the expression of *Lztr1* in the mouse telencephalon, a region of the brain that contains key brain structures necessary for higher executive function, including movement, learning, memory, and emotion (Rubenstein, 2011). We analyzed the late embryonic (E18.5) and postnatal (1 month) stages for *Lztr1* gene expression. *In situ* hybridization for *Lztr1* revealed enriched expression in the mantle zone of the developing cortex at the rostral and caudal levels in E18.5 embryos (Figures 1A,B). A weak expression was detected around the telencephalic ventricles that was more apparent at the rostral levels (Figure 1A). *Lztr1* expression was also detected outside of the telencephalon, in the dorsal thalamus of the diencephalon and the trigeminal nerve (Figure 1B). The expression of *Lztr1* showed a similar pattern in postnatal brains, with a widespread expression in the cortex at both levels (Figures 1C,D). In addition, we detected enriched expression in the basolateral amygdala complex and hippocampus (Figure 1D). The expression of *Lztr1* was also maintained in the mature thalamus of the diencephalon (Figure 1D). A recent report characterized a *Lztr1* KO mouse from the European conditional knockout consortium (Skarnes et al., 2011; Steklov et al., 2018; Sewduth et al., 2020); however, the lacZ expression, which is knocked into the *Lztr1* locus, was not analyzed. We also generated a variant of this line (see section “Methods”) and analyzed the lacZ activity and gene expression. LacZ activity was specific to *Lztr1^*lacZ/*+^* heterozygous mice (compare Figure 1E to Figure 1F). In addition, we detected a robust lacZ activity in the postnatal cortex, hippocampus, and thalamus in the same pattern as was detected with the *Lztr1* gene expression (Figure 1G). High magnification examination revealed isolated lacZ + cells in the corpus callosum where oligodendrocytes and astrocytes reside (Figure 1H). Given the isolated nature of the cells and the punctate appearance of lacZ + cells, we also performed *lacZ* gene expression and β-galactosidase immunohistochemistry, which revealed positive cells in the white matter (Figures 1I,J). We confirmed this result with high magnification of the *Lztr1* gene expression in the white matter (Figure 1K). The β-galactosidase antibody expression was the most robust method for detecting these cells, likely from the ABC signal amplification used during immunohistochemistry. Our expression analysis suggests that *Lztr1* expression is enriched in distinct cell types of the cortex, amygdala, hippocampus, and white matter in the telencephalon.

### Generation of a Telencephalon-Specific Knockout of *Lztr1*

**FIGURE 2:**
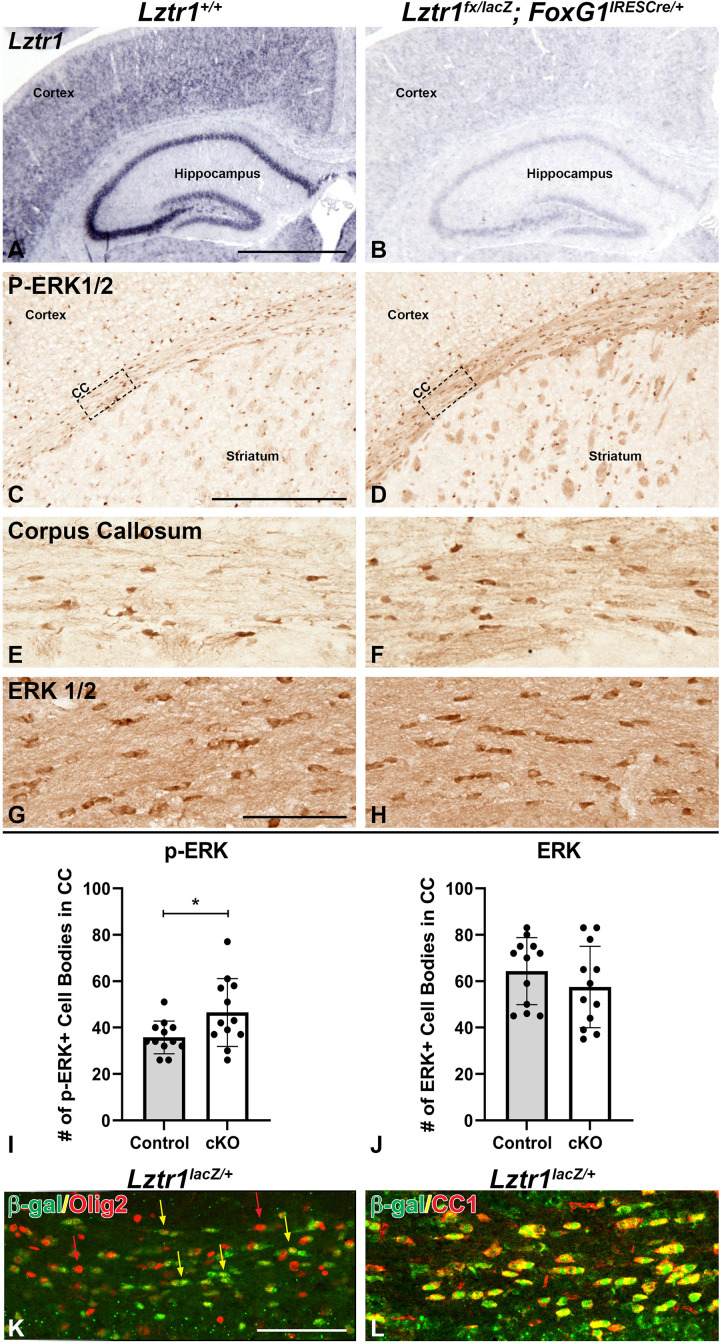
Conditional deletion of *Lztr1* using *FoxG1^*IRES*– *Cre/*+^* results in increased p-ERK1/2 expression in postnatal animals at postnatal day 21. *Lztr1* gene expression shows a reduction of expression in the cKO, especially in the cortex and hippocampus **(A,B)**. *Lztr1* cKO shows increased p-ERK1/2 expression **(D,F)** compared to controls **(C,E)**. Increased p-ERK1/2 expression is especially observable in major white matter tracts of the corpus callosum **(C–F)**. Total ERK1/2 staining is similar in *Lztr1* cKO compared to control in the corpus callosum **(G,H)**. Quantification of p-ERK1/2 and ERK1/2 by number of cell bodies per field in the CC **(I,J)**. β-gal reporter expression in *Lztr1^*lacZ/*+^* mice shows co-expression with oligodendrocyte lineage marker Olig2 **(K)** and mature oligodendrocyte marker CC1 **(L)**. Scale bars = 1 mm for **(A,B)**, 500 μm for **(C,D)**, and 100 μm for **(E–H,K,L)**. BLA, basolateral amygdala; CC, corpus callosum. The asterisk in **(I)** refers to a *p* < 0.05 as detected by Student’s *t*-test.

It has previously been shown that germline *Lztr1* KO does not survive to postnatal stages (Steklov et al., 2018; Sewduth et al., 2020). This limitation prevents modeling of the recessive hypomorphic or loss-of-function human mutations in organ systems, including the brain. Therefore, we established a conditional allele for *Lztr1* (Skarnes et al., 2011; Sewduth et al., 2020). We developed a strategy to model *Lztr1* deficiency during embryonic and postnatal development using an *Lztr1* conditional allele (see section “Methods”) and the *Foxg1^*IRES*–*cre/*+^* telencephalon-specific cre line (Kawaguchi et al., 2016). Unlike germline *Lztr1* mutants (Steklov et al., 2018), conditional mutants (*Lztr1^*fx/lacZ*^; Foxg1^*IRES*–*cre/*+^*) were viable to postnatal stages and showed reduced *Lztr1* expression in the cortex and hippocampus at P21 (compare Figure 2B to Figure 2A). Given that *Lztr1* is a RASopathy gene and implicated in regulating RAS and the MAPK pathway, we analyzed MAPK pathway activation in the brain using p-ERK1/2 expression. *Lztr1* cKOs showed an enhanced immunoreactivity for p-ERK1/2 in the corpus callosum of the white matter (compare Figures 2D,F to Figures 2C,E). This result was observed at all levels of the corpus callosum, including the lateral, medial, and caudal regions (Figures 2D,F and data not shown). The increase in staining was observed generally throughout the white matter and in distinct cell bodies. In fact, *Lztr1* cKOs exhibited a 30.1% increase in p-ERK1/2 + cell bodies within the white matter (compare Figure 2F to Figure 2E, graph in Figure 2I, *p* = 0.0319, *n* = 3). We did not detect a difference in total ERK1/2 immunoreactivity in the corpus callosum (Figures 2G,H, graph in Figure 2J), further supporting pathway activation using p-ERK1/2 expression in the Lztr1 cKO. Interestingly, we did not detect enhanced p-ERK1/2 immunoreactivity in the cortex despite a robust *Lztr1* expression in this region (Figure 1), which suggests a potential regional difference in pathway activation in *Lztr1* cKOs. However, this might be due to limitations of the immunohistochemistry technique requiring a certain threshold of change to view the difference. Based on the robust difference in the *Lztr1* cKO white matter, we performed immunofluorescence on *Lztr1* reporter mice with β-gal and oligodendrocyte markers. Olig2 is a marker of the entire oligodendrocyte lineage, and CC1 labels maturing oligodendrocytes during postnatal development (Emery and Lu, 2015). We found an overlap of β-gal cells with both Olig2 and CC1 (Figures 2K,L). While we detected a nearly complete overlap of β-gal with CC1, there were many Olig2 cells only (red arrows in Figure 2K), suggesting that Lztr1-driven β-gal is enriched in maturing oligodendrocyte but does not label the entire lineage. This is supported by online gene expression databases (Marques et al., 2016) which show that *Lztr1* is expressed in the entire oligodendrocyte lineage with a higher expression at mature stages (data not shown). Our results describe a genetic approach to study *Lztr1* deficiency in postnatal brain and suggest that *Lztr1* is required for normal control of MAPK pathway activation in the white matter regions.

### *Lztr1* Conditional Mutants Exhibit Changes in Glial Cell Marker Expression

**FIGURE 3:**
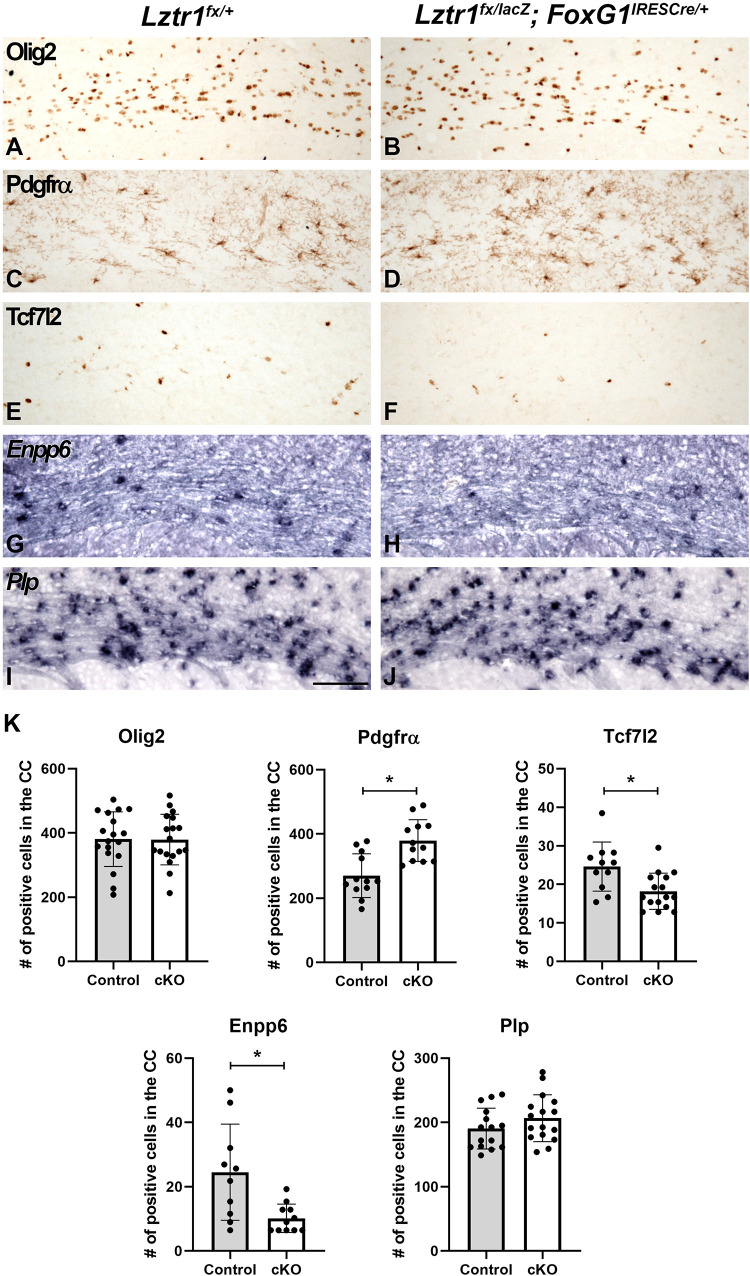
Loss of *Lztr1* impacts distinct stages of oligodendrocyte maturation in the corpus callosum of postnatal animals at 1 month of age. Olig2 marks the entire oligodendrocyte lineage, and no difference between the number of Olig2 + cells is observed in controls **(A)** and cKOs **(B)**. The OPC marker Pdgfrα was increased in the corpus callosum in cKOs **(D)** compared to controls **(C)**. Tcf7l2 and *Enpp6* expression observed in differentiating and newly formed oligodendrocytes **(E,G)** are decreased in cKOs **(F,H)**. The mature oligodendrocyte marker *Plp* appears similar in controls and cKOs **(I,J)**. Quantification of oligodendrocyte markers by the number of positive cells per field in the CC **(K)**. Scale bar = 100 μm for **(A–J)**. The asterisk in **(K)** refers to a *p* < 0.05 as detected by Student’s *t*-test.

Previous work has shown that the MAPK pathway activation plays a key role at multiple stages of oligodendrocyte development (Li et al., 2012; Fyffe-Maricich et al., 2013; Ishii et al., 2013; Li et al., 2014). In addition, RASopathy mutations have been shown to impact both early and mature stages of oligodendrogenesis (Bennett et al., 2003; Dasgupta and Gutmann, 2005; Zhu et al., 2005; Mayes et al., 2013; Ehrman et al., 2014; Lopez-Juarez et al., 2017; Titus et al., 2017; Aoidi et al., 2018; Holter et al., 2019). To determine if *Lztr1* plays a role in oligodendrocyte development, we analyzed stage-specific markers that label oligodendrocyte progression at 1 month of age when developmental myelination is largely complete. We did not detect dramatic changes in Olig2 and *Plp* gene expression which labels the entire lineage and maturing oligodendrocytes, respectively (Figures 3A,B,I,J,K). However, *Lztr1* cKOs exhibited alterations in stage-specific markers that label distinct transition points from progenitors to differentiated oligodendrocytes. Pdgfrα labels OPCs and is increased in the *Lztr1* cKO corpus callosum (Figures 3C,D,K; 40.5% increase, *p* = 0.000581, *n* = 3). This is in line with other RASopathy mouse models that show increased OPC markers (Bennett et al., 2003; Dasgupta and Gutmann, 2005; Ehrman et al., 2014; Holter et al., 2019). Tcf7l2 is a key factor in oligodendrocyte differentiation (Fancy et al., 2009; Ye et al., 2009; Hammond et al., 2015; Zhao et al., 2016). *Lztr1* cKOs show a reduction in Tcf7l2 + cells in the corpus callosum (Figures 3E,F,K; 26.0% decrease, *p* = 0.00587, *n* = 3). *Enpp6* is a transient marker enriched in newly formed oligodendrocytes (Zhang et al., 2014; Xiao et al., 2016). Similar to Tcf7l2 expression, *Lztr1* cKOs also showed a reduction in *Enpp6* expression in the white matter (Figures 3G,H,K; 58.6% decrease, *p* = 0.006622, *n* = 3). These results suggest that *Lztr1* deficiency results in changes in the stage-specific expression of oligodendrocyte markers, with increased OPCs and decreased expression of markers that label newly formed oligodendrocytes. Despite this, the total oligodendrocytes labeled by Olig2 and mature oligodendrocytes labeled by *Plp* were not affected.

**FIGURE 4:**
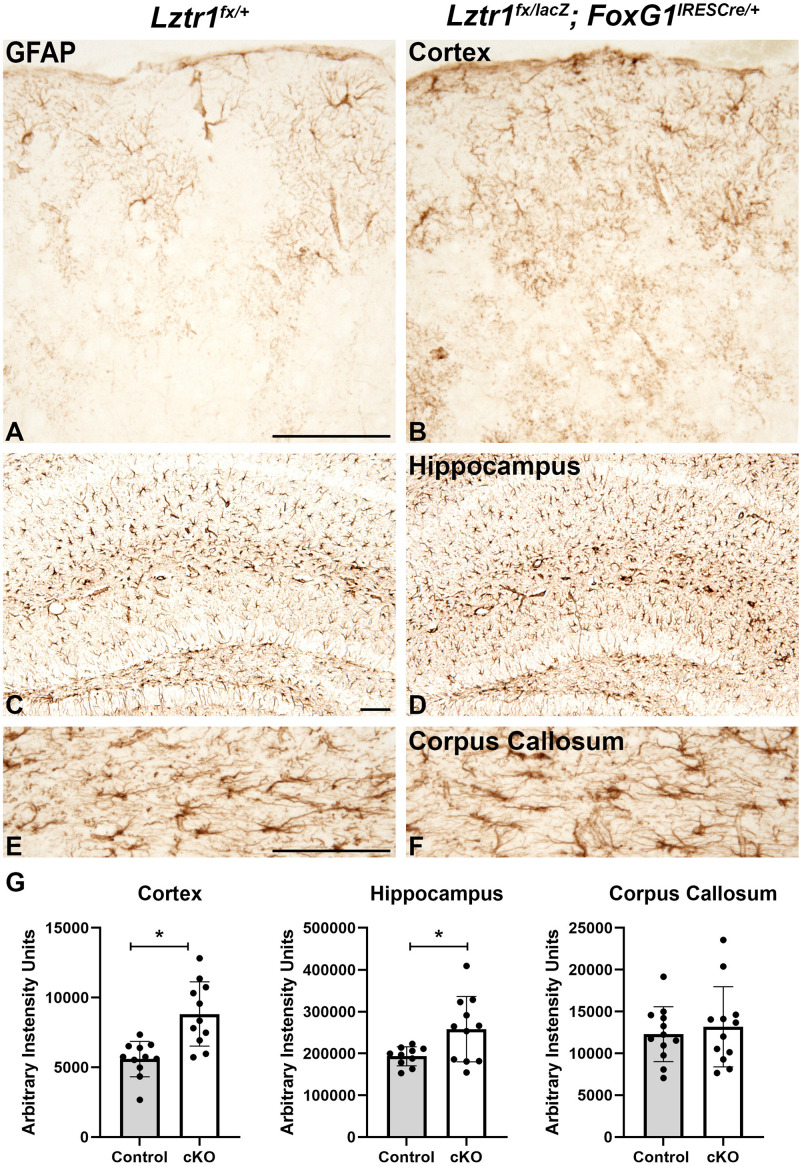
Loss of *Lztr1* increases the expression of the astrocyte marker GFAP in postnatal animals at 1 month of age. Increased GFAP as measured by stain intensity (arbitrary intensity units) is observed in the cortex and hippocampus of cKOs compared to controls **(A–D,G)**. The GFAP expression in the corpus callosum was similar in cKOs compared to controls **(E,F)**. Quantification **(G)**. Scale bars = 100 μm for **(A–F)**. The asterisk in **(G)** refers to a *p* < 0.05 as detected by Student’s *t*-test.

In addition to oligodendrocytes, the white matter also contains astrocytes (reviewed in Molofsky et al., 2012). In control brains, GFAP is robustly expressed in the white matter and hippocampus but only in isolated cells of the cortex (Figures 4A,C,E). *Lztr1* cKOs showed increased GFAP-positive cells in the cortex and hippocampus (Figures 4A–D,G). The increase was measured by total pixel intensity in the lateral cortex and CA region of the hippocampus. There was a 57.8% increase in the cortex (*p* = 0.000596, *n* = 3) and a 33.5% increase in the hippocampus (*p* = 0.0208, *n* = 3). The GFAP-positive cells in the corpus callosum appeared to have more defined cell bodies in the *Lztr1* cKOs compared to controls (Figures 4E,F). However, the staining intensity was not significantly different in the corpus callosum, indicating that the robust increase in astrocytes might be associated more with gray matter areas. GFAP expression labels normal astrocytes but is also expressed in reactive astrocytes in injury and disease models. The increase of GFAP immunoreactivity in the cortex is supported by previous reports that loss of the RASopathy gene *NF1* results in a robust increase in GFAP expression in reactive astrocytes (Hewett et al., 1995; Nordlund et al., 1995; Gutmann et al., 1999; Rizvi et al., 1999). This suggests that hyperactivation of the RAS/MAPK pathway through *NF1* or *Lztr1* loss results in increased GFAP + astrocytes. Overall, our results from telencephalon-specific conditional knockout suggest that *Lztr1* plays a role during oligodendrocyte and astrocyte development in the postnatal telencephalon.

## Discussion

In this study, we evaluated *Lztr1* in the developing brain by characterizing the expression in the telencephalon. Our results indicate that *Lztr1* is highly expressed in the embryonic and postnatal cortex. We detected robust expression in the postnatal hippocampus, amygdala, and oligodendrocytes. To address a role for *Lztr1* in the postnatal brain, we developed a conditional approach to specifically delete *Lztr1* in the telencephalon using *Foxg1^*IRES*–*cre/*+^* and an *Lztr1* conditional allele. We found that *Lztr1* deletion results in robust MAPK pathway activation and impacts the oligodendrocyte lineage. In addition, we detected ectopic GFAP astrocyte expression in the cortex of conditional mutants. Our results suggest that *Lztr1* plays a role in MAPK pathway activation and normal glial cell development in the telencephalon. These findings provide a “roadmap” of *Lztr1*-enriched areas of the brain that might be affected by *Lztr1* mutation or deficiency. In addition, we describe a new genetic approach to address the impact of *Lztr1* deficiency in the brain and model loss-of-function mutations.

The expression of *Lztr1* has been described as widespread (Kurahashi et al., 1995). However, expression data on tissue sections of any organ system, including the brain, have not been reported. Therefore, detecting enriched *Lztr1* expression in the cortex, hippocampus, amygdala, and oligodendrocytes is significant. The expression data, combined with the results from our telencephalon-specific conditional mutant showing elevated MAPK pathway activation in the white matter, provide important clues to distinct cell populations impacted by *Lztr1* mutations. Indeed previous efforts have not been able to describe the postnatal impact of *Lztr1* mutations since germline loss resulted in lethality before birth from severe cardiovascular defects (Steklov et al., 2018; Sewduth et al., 2020), thereby preventing a postnatal analysis of the brain. Our strategy is the first to address the requirement of *Lztr1* in the postnatal brain. Given the high cortical expression of *Lztr1*, we were surprised in the lack of widespread MAPK pathway activation in the cortex of conditional mutants. However, this may be a result of the immunohistochemistry technique on tissue sections requiring a threshold of pathway activation to detect the increase. Alternatively, the loss of *Lztr1* might regulate other RAS-regulated pathways such as AKT/mTOR in the cortex or white matter. In addition, it remains unknown if *Lztr1* loss might impact specific cortical cell populations, neural activity, or neuronal function and behaviors. Our conditional mutant approach will provide a good opportunity to address these issues. In addition, future experiments to parse cortical-specific vs. oligodendrocyte-specific requirements for *Lztr1* will be important for addressing cell type-specific impact on neuronal activity or function, oligodendrocyte myelination, and behaviors. Previous work has elucidated specific behaviors impacted by RAF1 and NF1 RASopathy mutations (Lopez-Juarez et al., 2017; Holter et al., 2019). It will be interesting to compare how these behaviors are impacted in an *Lztr1*-deficient mouse.

There exist multiple lines of evidence showing that appropriate regulation of RAS signaling and the MAPK pathway is crucial for normal oligodendrocyte development. Evidence from RASopathy animal models show that the dysregulation of the RAS/MAPK pathway has a robust effect on early progenitor neural stem cells or OPCs (Bennett et al., 2003; Dasgupta and Gutmann, 2005; Zhu et al., 2005; Wang et al., 2012; Ehrman et al., 2014; Li et al., 2014; Sun et al., 2015; Holter et al., 2019). Hence, our data which indicate that *Lztr1* loss increases OPC populations is in line with other studies on RASopathy mutations at various levels of the pathway. Previous studies have utilized hyperactivation of MEK to show that a normal level of MAPK signaling is crucial at the late stages of oligodendrocyte development and myelination (Fyffe-Maricich et al., 2013; Ishii et al., 2013). In addition, *Nf1* loss or *HRAS-G12V* RASopathy mutations have also been shown to impact mature and myelinating oligodendrocytes (Mayes et al., 2013; Lopez-Juarez et al., 2017; Titus et al., 2017). Therefore, the reduction in the newly formed oligodendrocyte markers observed in the *Lztr1* cKO might be from either a role for *Lztr1* specifically during this mature stage or a secondary effect of the increased OPC populations that do not transition to the newly formed oligodendrocyte stage in a normal manner. Either result suggests that *Lztr1* function is key to oligodendrogenesis. However, future experiments will be important to define if developmental deletion of *Lztr1* impacts oligodendrocyte myelination and subsequently results in any behavioral phenotypes. It will also be interesting to parse stage-specific roles for *Lztr1* in either OPCs or mature oligodendrocytes and also reveal downstream signaling mechanisms underlying the oligodendrocyte phenotype.

GFAP is one of the most common markers for astrocytes in the postnatal mouse brain, but it does not label all astrocytes and is also a marker of reactive astrocytes after various injuries or defects (reviewed in Molofsky et al., 2012). An increased GFAP expression in reactive astrocytes was identified in *NF1* mutant tissue (Hewett et al., 1995; Nordlund et al., 1995; Gutmann et al., 1999, 2001; Rizvi et al., 1999; Hegedus et al., 2007). A recent report identified that a mouse model for the Noonan syndrome mutation *RAF1-L613V* exhibits an increased GFAP expression in the cortex and hippocampus (Holter et al., 2019). We observed a similar effect in the *Lztr1* cKO cortex and hippocampus. However, it should be noted that we analyzed our tissue after the weaning stage (1 month), whereas the study of Holter et al. (2019) examined 2-month-old animals. It will be interesting to determine how the increase in GFAP + cells changes during development and the late adult stages in *Lztr1* cKOs. Interestingly, a previous study using activating HRAS mutations has shown that iPSC-derived astrocytes from HRAS-G12S Costello patients and astrocytes from HRAS-G12V mice undergo precocious maturation, suggesting that dysregulation of the RAS/MAPK pathway can affect the timing of astrocyte development and even impact neuronal populations (Krencik et al., 2015). Therefore, it may be that *Lztr1* gene function is crucial in both astrocyte and oligodendrocyte development. Alternatively, the astrocyte phenotype may be a readout of the oligodendrocyte defect or an unknown defect in cortical neurons or activity promoting reactive astrogliosis. Future experiments in which *Lztr1* is conditionally inactivated in either the astrocyte or oligodendrocyte lineage will address this issue.

Increasing genetic evidence in 22q11.2 deletion syndrome, NS, and glioma tumors point toward the negative health impact of *Lztr1* mutation or deletion in multiple organ systems, including the brain (Kurahashi et al., 1995; Frattini et al., 2013; Yamamoto et al., 2015; Johnston et al., 2018). Therefore, studies using animal models are key to understanding the role of *Lztr1* in specific cells of the brain. Our results identify enriched regions of *Lztr1* expression and describe a new conditional mutant approach to determine what cells are most susceptible to *Lztr1* dysregulated signaling in the telencephalon. Our expression results and new mouse model are important first steps in understanding *Lztr1* human disease in the brain.

## Data Availability Statement

The original contributions presented in the study are included in the article, further inquires can be directed to the corresponding author.

## Ethics Statement

The animal study was reviewed and approved by the Cincinnati Children’s Hospital Medical Center Institutional Animal Care and Use Committee.

## Author Contributions

RW, CP, and MT performed the conception and experimental design. MT, NS, and DN performed the data collection, analysis, and/or animal work. RW and MT wrote the manuscript. CP, LE, and DN edited the manuscript and provided critical input to the final manuscript. All authors contributed to the article and approved the manuscript.

## Conflict of Interest

The authors declare that the research was conducted in the absence of any commercial or financial relationships that could be construed as a potential conflict of interest.
